# Impact of an asynchronous telerehabilitation program on the self-efficacy and motivation for physical activity in discharged COVID-19 patients: a secondary analysis of a randomized controlled trial

**DOI:** 10.1080/07853890.2025.2573148

**Published:** 2025-10-25

**Authors:** Beatriz Carpallo-Porcar, Carolina Jiménez-Sánchez, Irene Liñares-Varela, Laura Bafaluy-Franch, Paula Córdova-Alegre, Sara Pérez-Palomares, Manuel Gómez-Barrera, Sandra Calvo

**Affiliations:** ^a^ Department of Physical Therapy, Faculty of Health Sciences, Universidad San Jorge, Zaragoza, Spain; ^b^ Instituto de Investigación Sanitaria de Aragon (IISAragón), Zaragoza, Spain; ^c^ Department of Physiatry and Nursing, Faculty of Health Sciences, Zaragoza, Spain; ^d^ Departament of Pharmacy, Faculty of Health Sciences, Universidad San Jorge, Zaragoza, Spain; ^e^ Pharmacoeconomics and Outcomes Iberia, Madrid, Spain.

**Keywords:** Adherence, COVID-19, motivation, psychological, physical therapy, self-efficacy, telerehabilitation

## Abstract

**Background:**

Telerehabilitation has become an important tool for the recovery of coronavirus disease (COVID-19) patients, allowing treatment to be continued remotely for this and other pathologies. Self-efficacy plays a key role in motivating and ensuring adherence to these programs. The aim of this study was to evaluate the effects on self-efficacy (GSES) and sport motivation (BRSQ-36) and analyze the correlation between self-efficacy and physical condition after a program composed of therapeutic exercises and education.

**Materials and methods:**

This pilot randomized controlled trial included 35 post-discharge COVID-19, with two groups: an asynchronous telerehabilitation and a booklet-based rehabilitation groups who undertook a 12-week intervention of therapeutic exercise and education..

**Results:**

The telerehabilitation group showed better results in all variables analyzed, with moderate and large clinical changes in overall motivation *d* = 0.8, but no significant changes. At the 3- and 6-month follow-up, statistically significant differences in self-efficacy were found in favor of the telerehabilitation group (3-m, *p* = 0.025, *d* = 0.76); 6-m, *p* = 0.007, *d* = 0.79). The telerehabilitation group showed better results in ‘Extrinsic Motivation’ (3-m, *p* = 0.037, *d* = 0.75; 6-m, *p* = 0.010, *d* = 0.94) and ‘Identified Regulation’ (3-m, *p* < 0.001, *d* = 1.09; 6-m, *p* = 0.005, *d* = 0.49) after 3- and 6-month follow-up. . In all patients, a direct correlation was found between self-efficacy and meters walked in the six minutes’ walk test (6 MWT) (*p* = 0.022; *R*^2^ = 0.149), ‘30 STST and 30’ ACT (*p* = 0.002; *R*^2^ = 0.261; *p* = 0.017; *R*^2^ = 0.160), respectively; an inverse correlation was found after three months with the fatigue variable (*p* < 0.001; *R*^2^ = 0.2858) and after six months (*p* < 0.001; *R*^2^ = 0.2889).

**Conclusions:**

The findings highlight the potential of asynchronous telerehabilitation to improve self-efficacy and extrinsic motivation in a short period of time, which could facilitate better adherence to rehabilitation programs and improve physical condition. However, the results seem to be limited in the long term.

## Introduction

Spain was one of the main European hotspots of the new betacoronavirus, (SARS-CoV-2) due to the speed of the spread within its borders. During the first wave of the pandemic the average number of cases was 577.8 per 100,000 inhabitants, with a hospitalization rate of 232.8 per 100,000 inhabitants [1]. The most common reported post-discharge symptoms and rehabilitation needs are new illness‐related fatigue (64%) and breathlessness (50%), along with a clinically significant drop in health-related quality of life (53%); 61,4% presented with comorbidities and 16.5% of patients had a moderate or severe degree of dependence for activities of daily living [2].

Telemedicine has proliferated to overcoming geographic barriers, especially for people with less access to healthcare services, from rural areas, or who experience any mobility limitations [2]. Given the high pressure on healthcare, mandatory social distancing, and comorbidity/vulnerability of COVID-19 survivors, traditional face-to-face rehabilitation care became unfeasible. Telerehabilitation after hospital discharge provided a therapeutic window. Although the telerehabilitation models used during the acute post-hospitalization phase varied, they consistently demonstrated improvements in patients’ physical function [3–5]. The recommended physical therapy treatments are home-based programs that focus on breathing exercises, strength training, resistance, and aerobic activities [6,7]. However, with independent rehabilitation at home, there is a risk that patients may struggle with adherence and motivation, which can potentially impair the effectiveness of therapy [8].

Self-efficacy is defined as an individual’s own belief in their ability to perform a behavior or control a specific outcome [9]. Self-efficacy, included as an explanation of motivational behavior, is a reliable predictor of adherence to different health behaviors, such as medication adherence, nutrition, or participation in physical exercise [10,11]. Therefore, it is considered the main determinant of consistent levels of physical activity, specifically for the continuity of a home exercise program [12].

Self-efficacy has been demonstrated to be particularly relevant in telerehabilitation in individuals with pulmonary [13,14] and cardiovascular diseases [15,16], as it enhances satisfaction, confidence, motivation, and adherence. These improvements in self-efficacy may be attributed to the empowering effect of technology, even among patients who have never used a computer before [14]. However, self-efficacy is not an inherent personal trait; it is influenced by various factors and situations. Confidence in one’s own ability to exercise, for example, can vary significantly under challenging conditions, such as when facing exercise barriers (exercise barrier self-efficacy) [10,17]. The circumstances created by the pandemic, characterized by high morbidity, mortality, and social isolation, have heightened social vulnerability. This environment, in which external circumstances and community factors increase the risk of negative outcomes, has led to a decline in collective efficacy [18].

Motivation is the internal or external force that acts on an individual to trigger, direct or sustain behavior; it allows us to create habits, try new things, and sustain effort in some tasks that we consider rewarding or productive [19]. Special attention is paid to motivation in chronic diseases that require long-term rehabilitation, where it is important to maintain patient involvement [20]. In cardiac telerehabilitation, the use of remote self-monitoring equipment has been confirmed to enhance patients’ motivation for behavioral change [21]. Videoconferencing promotes the relationship between patients and therapists, knowledge, and motivation for rehabilitation in orthopedic patients [22]. However, the motivation is of special interest in neurorehabilitation; the use of telerehabilitation systems enhances patient engagement by motivating them to conduct their rehabilitation training at home [23].

Both self-efficacy and motivation are factors that can contribute to the follow-up of rehabilitation programs and, therefore, to physical therapy outcomes. To our knowledge, there is no study about self-efficacy or motivation during pandemic time in discharged COVID-19 patients who have undergone telerehabilitation. As healthcare systems increasingly adopt remote care models, understanding psychological factors that influence patient engagement such as, self-efficacy and motivation have become essential. These variables are relevant not only to post-pandemic recovery but also to the broader implementation of digital rehabilitation strategies for chronic and post-acute conditions [24].

Self-efficacy and motivation have been shown to play a critical role in patient adherence and outcomes across various pathologies, including stroke, cardiovascular disease, COPD, cancer-related fatigue, and musculoskeletal disorders. Incorporating these psychosocial dimensions into telerehabilitation programs can enhance treatment personalization, improve adherence, and optimize recovery trajectories [13]. Therefore, the aim of this randomized, controlled, single-blind pilot study was to evaluate the effects of a multimodal telerehabilitation program compared to a booklet-based rehabilitation approach on self-efficacy and physical activity motivation in discharged COVID-19 patients. Additionally, the study sought to examine the relationship between self-efficacy and the other physical variables, aiming to identify potential interactions that may enhance the understanding of its impact on overall study outcomes.

## Materials and methods

### Study design

A secondary analysis of psychosocial variables was performed following a previous pilot randomized controlled trial with two parallel intervention arms: the asynchronous telerehabilitation group (ATG) and booklet-based rehabilitation group (BRG). The study followed the CONSORT (Consolidated Standards of Reporting Trials) extension for randomized pilot studies. This study was approved by the Ethics Committee of Aragón (PI21/019) and was registered at clinicaltrials.gov (NCT04794036).

The study lasted 20 months, with a 12-week intervention and two follow-ups at 3 and 6 months. Recruitment began in April 2021 and the last follow-up was conducted in December 2022. The full protocol for this study and the primary and other secondary results have been previously published [25–27].

### Participants

Patients from the two hospitals were invited to participate in this pilot study once they were discharged after COVID-19. Recruitment was conducted in the first month after discharge by the post-COVID-19 Rehabilitation Unit’s physician from two Spanish hospitals.

Potential participants were informed of the study characteristics, and those who wanted to participate and met the following criteria were enrolled. The inclusion criteria were as follows: (1) patients in the post-acute phase of COVID-19; (2) patients admitted to the hospital for at least 5 days for COVID-19; (3) aged 18–75 years; (4) independent standing with or without technical aids; and (5) fatigue severity score of ≥4 points on the Fatigue Severity Scale[28]. The exclusion criteria were as follows: (1) any other central and/or peripheral neurological disorders; (2) a previous history of rheumatic pathology or acute musculoskeletal injury; (3) severe hypoxemia at the moment of inclusion, defined as oxygen saturation below 90% or a respiratory rate ≥30; (4) any unstable cardiac comorbidities or signs of cardiovascular instability such as uncontrolled arrhythmias, blood pressure, and/or effort angina; (5) any other contraindicated pathology for moderate-intensity aerobic or strength exercise; (6) a score ≤24 evaluated with the validated Spanish version of the Mini-mental State Examination (MMSE-MEC) [29]; (7) no daily access to the internet; and 8) not being able to follow oral and written instructions in the Spanish language.

Participants were randomized to the ATG or BRG in a 1:1 ratio *via* the software www.randomizer.org by an independent researcher using the software www. randomizer. org. The same evaluator performed all measurements and was blinded to the randomization.

### Procedure

Volunteers who met the inclusion criteria signed a consent form. The assessments were performed at the hospital by the same blinded physical therapist. All participants were assessed at four points during the study: at baseline and at the end of the intervention in a face-to-face manner, and at the 3- and 6-month follow-ups by telephone.

The researcher responsible for the intervention installed the telerehabilitation platform on the mobile device (*via* an app) of the ATG participants and explained the home booklet-based rehabilitation to BRG participants. The onset and progression of the therapeutic exercise program were determined based on the fatigue perceived by each patient.

During the three-month period, control phone calls to each participant were performed every two weeks to personalize the rehabilitation program, ensure the absence of adverse effects, and support treatment adherence in both groups.

### Intervention

The intervention was the same in both groups. It consists of a home-based 12-week multimodal program. It is characterized by therapeutic exercise (TE) designed following the main recommendations for these patients in 2020 [30–32]. Health education (HE) was developed by the research team following scientific recommendations to support patients’ self-management, adaptation to treatment, and their disease.

The TE program included aerobics, strength resistance, and breathing exercises for three days a week [33]. These were divided into three progressive levels of intensity. The lung capacity exercises were the same throughout the intervention and were performed daily. This program was designed to complement HE, as therapeutic exercise influences not only physical condition but also psychosocial components.

The HE programme consisted of three blocks of health advice. The first block contains recommendations for the prevention of new infections. The second focused on reducing the effects of isolation and social distancing, as this could increase the psychological symptoms of these patients. The third block was composed of advice on the self-management of psychological symptoms.

#### Intervention group: ATG

ATG performed the multimodal program *via* a telerehabilitation platform on the Internet (www.hefora.net) or *via* a mobile app (HEFORA, Fisio Consultores, Zaragoza, Spain). The TE program was presented in the form of explanatory videos with specific descriptions. The platform allowed the physical therapist to customize the number of sets, repetitions, speeds, and observations for each patient. The TE program was presented in the form of animated educational videos, in which health and emotional tips were explained to patients.

#### Control group: BRG

Participants in the BRG received the multimodal program through booklet-based rehabilitation that contained the main pictures and descriptions for each exercise at each level. The exercise series, repetitions, and recommended rest were individualized in biweekly control phone calls. In addition, the patients in the BRG underwent the same TE program as the patients in the ATG, but in text form **(Suppl 1)**.

### Outcome measures

Demographic data, including age, sex, weight, body mass index (BMI), tobacco consumption, level of physical activity, days and type of hospital stay, and the type of rehabilitation received were recorded at baseline. Physical status: post-COVID functional scale (PCFS), six minutes’ walk test (6 MWT), −30 s sit to Stand Test and Arm Curl Test (STST and ACT) from the primary study [26] was used for correlations.

PCFS is a scale that measures the functional limitations of patients discharged from hospital after COVID-19 ranging from 0= no limitations to 4 = severe limitations [34]. The 6 MWT measures aerobic and gait capacity and the STST and ACT measure upper and lower limb strength.

Self-efficacy was measured using the General Self-Efficacy Scale (GSES) developed by Schwarzer and adapted to Spanish [35,36]. It comprises 10 items scored on a Likert scale ranging from 1 (strongly disagree) to 5 (strongly agree), with a range from 10 to 50. The higher the score, the greater the perceived self-efficacy. The Behavioral Regulation in Sport Questionnaire (BRSQ-36) [37] was used to measure the motivation to practice physical activity. It has been shown to be reliable and adaptable to Spanish [37,38]. The BRSQ-36 is composed of 36 items and is divided into six main domains in three groups: 1) Amotivation (AM) (items 9,18,27,36). 2) Extrinsic motivation (EM): external regulation (EX) (items 8,17,26,35); introjected regulation (IJ) (items 7,16,25,34); identified regulation (ID) (items 6,15,24,33); and integrated regulation (IG) (items 5,14,23,32). 3) Intrinsic motivation: Knowledge (IM-K) (items 2,11,20,29), accomplish (IM-A) (items 4,13,22,31), stimulation (IM-S) (items 3,12,21,30) and general (IM-G) (items 1,10,19,28). The items were rated on a Likert scale ranging from 1 (not at all true) to 7 (very true). A higher total score for each factor indicates higher dominance of that motivational factor.

### Statistical analysis

Data were analyzed using SPSS 28.0 (SPSS Inc, Chicago, IL). Descriptive statistics, including frequency counts for categorical outcomes, and measurements of central tendency and dispersion for continuous outcomes (standard deviation, 95% confidence interval) were calculated. The Shapiro–Wilk test was used to determine data normality. Intention-to-treat analysis was also performed. For the comparison of means between groups at each time point [2 groups × (pre, post, 3, and 6 months)], *t* tests for independent samples, Levene tests for parametric data, and Mann–Whitney *U* tests for non-parametric data were used. Friedman and Wilcoxon tests were performed to compare the intervention effects (Group × time [pre-intervention vs. post-intervention vs. follow-up 1 and 2]) on the outcomes due to the non-parametric distribution of the data. In the Wilcoxon test, type I error was divided by the number of tests performed. Chi-square, Fisher, and Fisher–Freeman–Halton tests of independence were used for categorical data. The significance level was set at *p* < 0.05 for all statistical analyses. The effect size and clinical significance were calculated using Cohen’s d: insignificant, small, medium, and large differences were reflected in effect sizes of <0.2, 0.2–0.5, 0.5–0.8 and >0.8, respectively. An ITT was performed to analyze the missing values. Linear regression was performed to determine the relationship between self-efficacy and the rest of the variables in the entire sample. A sample size of 50 participants (20 participants per arm) was established to answer the questions of this pilot study based on the sample size recommendations for this type of pilot clinical trial study [39–41].

## Results

**Figure 1.**
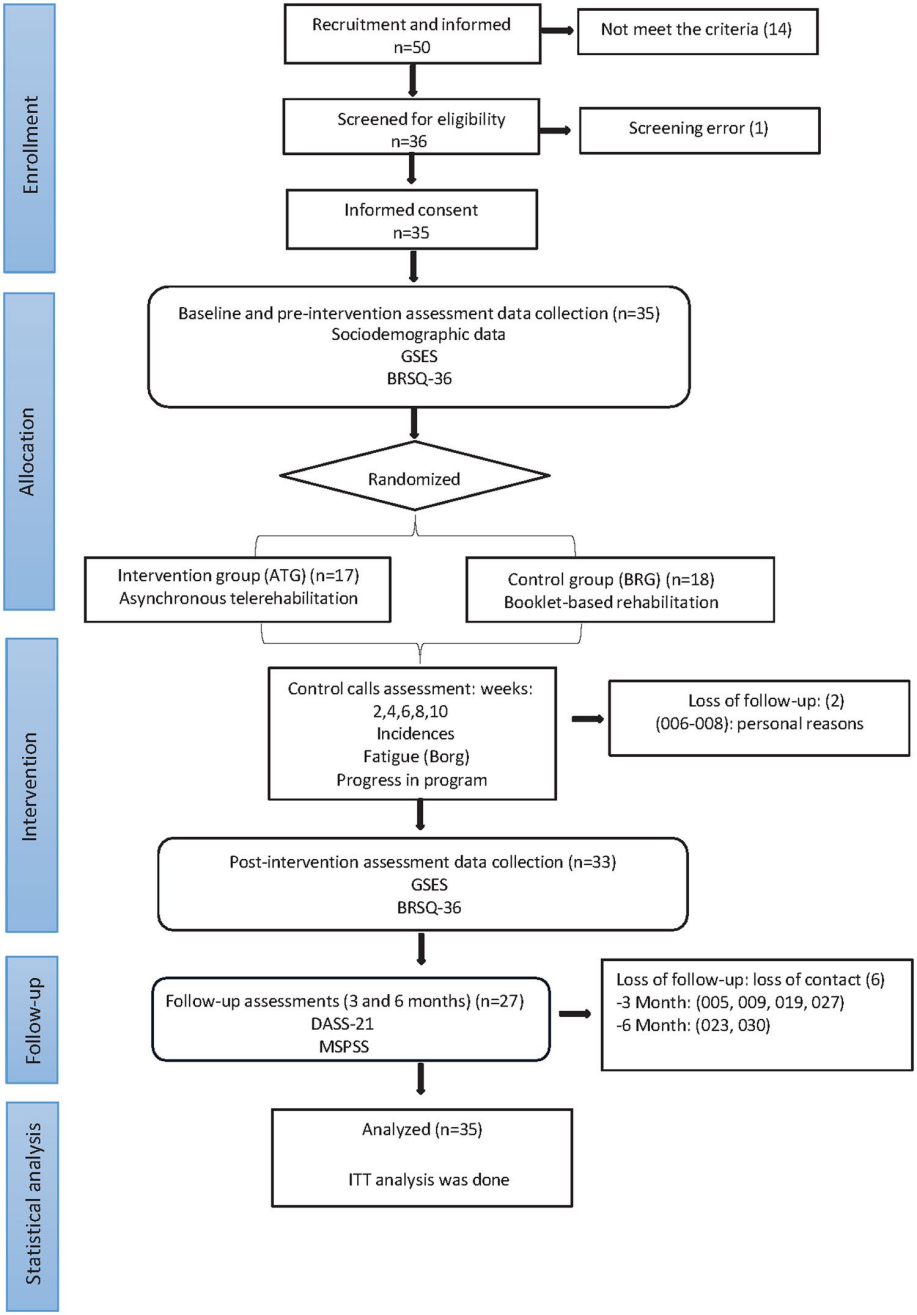
Flow chart of the study.

35 post-discharge COVID-19 patients were recruited, from February 2021 to March 2022. Figure 1 shows the study flow of enrollment.

Of the total sample, 57.1% were women with a mean (±SD) age of 58 (±8) years, who were overweight and sedentary at baseline. The participants were hospitalized for a mean average of 25 days, and 31.4% were intubated in the intensive care unit and ward. The fatigue severity was 5.98 points, and 45.7% had some functional limitations according to their post-COVID-19 Functional Status. At the beginning of the study, the ATG and BRG groups presented similar characteristics (*p* ≥ 0.05) in terms of sociodemographic variables, dates of admission, fatigue, and self-efficacy. With regard to BRSQ-36, the ATG showed a higher total score of the scale and the total score of extrinsic motivation due to differences in ‘integrated regulation.’ Both groups were similar in two other main domains: ‘internal motivation’ and ‘amotivation.’ All the data are presented in Table 1.

**Table 1.** Baseline characteristics.

		**Total *n* = 35**	**ATG *n* = 17**	**BRG *n* = *18***	***p* value**
***Sociodemographic***					
*Sex*	*n* (%)				0.380^P^
Men		15 (42.90)	6 (40.00)	9 (60.00)	
*Women*		20 (57.10)	11 (55.00)	9 (45.00)	
*Years*	*m* (SD)	58.00 (8.00)	58.00 (2.00)	59.00 (9.00)	0.543^T^
*BMI (kg/m^2^)*	*m* (SD)	28.89 (4.90)	28.75 (5.01)	29.03 (4.86)	0.869^T^
*Physical Activity (yes)*	*n* (%)	21 (60.00)	10 (47.60)	11 (52.40)	0.890^P^
*Activity Level (h/w)*	*m* (SD)	5.00 (3.30)	4.40 (3.00)	5.50 (3.50)	0.455^T^
*Smoker (Yes)*	*n*%	2 (5.70)	0 (0.00)	2 (100)	0.486^F^
***Dates of admission***					
*Days of admission*	*m* (SD)	25.00 (20.00)	24 (20.00)	25 (21.00)	0.812^T^
*Type of hospital stay*	*n* (%)				0.632^P^
Ward		24 (68.60)	11 (45.80)	13 (54.20)	
Ward and ICU		11 (31.40)	6 (54.50)	5 (45.50)	
*Oxigenotherapy*	*n* (%)				0.752^F^
None		15 (42.90)	6 (40.00)	9 (60.00)	
Nasal mask		9 (25.70)	5 (55.60)	4 (44.40)	
Intubation		11 (31.40)	6 (54.50)	5 (45.50)	
*Hospital rehabilitation (yes)*	*n* (%)	18 (51.40)	10 (55.60)	8 (58.80)	0.395^P^
***Post-discharge fatigue***					
*PCFS*	*n* (%)				0.118^F^
Some symptoms		10 (28.60)	2 (20.00)	8 (80.00)	
Some limitation		16 (45.70)	9 (56.30)	7 (38.90)	
Limitations for IADL		8 (22.90)	5 (62.50)	3 (37.50)	
Limitations for BADL		1 (2.90)	1 (100.00)	0 (0.00)	
*Fatigue (FSS)*	*m* (SD)	5.98 (0.89)	6.26 (0.68)	5.71 (1.01)	0.102^U^
*Fatigue level*	*n* (%)				0.118^F^
Low severity		6 (17.10)	1 (16.70)	5 (83.30)	
Moderated severity		8 (22.90)	3 (37.50)	5 (62.50)	
High severity		21 (60.00)	13 (61.90)	8 (38.10)	
***Psychosocial scales***					
*Self-efficacy (GSES)*	*m* (SD)	38.69 (8.47)	40.06 (9.55)	37.39 (7.35)	0.186^U^
*Physical activity motivation (BRSQ-36)*	*m* (SD)	153.95 (31.62)	167.24 (18.79)	141.4 (36.38)	0.011^U^
Extrinsic motivation		57.97 (11.03)	62.81 (6.37)	53.38 (12.74)	0.004^U^
Integrated regulation		20.27 (6.25)	23.25 (2.79)	17.55 (6.89)	0.007^U^
Identified regulation		22.57 (4.42)	24.43 (5.15)	21.11 (5.14)	0.092^U^
Introjected regulation		9.71 (5.06)	9.87 (4.06)	10.05 (5.89)	0.749^U^
External regulation		4.95 (1.62)	5.25 (1.80)	4.66 (1.32)	0.073^U^
Intrinsic motivation		96.22 (5.86)	96.44 (11.09)	84.89 (27.65)	0.641^U^
Knowledge		20.85 (6.76)	22.75 (5.04)	19.66 (7.97)	0.348^U^
Stimulation		22.08 (5.98)	24.19 (2.40)	20.17 (7.50)	0.180^U^
Accomplish		23.51 (4.15)	24.68 (2.57)	22.39 (4.94)	0.243^U^
General		23.76 (6.58)	24.81 (3.31)	22.66 (8.57)	0.622^U^
Amotivation		6.02 (3.55)	5.50 (1.89)	6.44 (4.59)	0.657^U^

BMI (kg/m^2^) = body mass index; ICU = intensive care unit; PCFS = post-covid functional scale; FSS = fatigue severity scale. GSES = General Self-Efficacy Scale. DASS-21 = Depression, Anxiety and Stress Scale; MSPSS = Multidimensional Scale of Perceived Social Support. BRSQ-36 = Behavioral Regulation in Sport Questionnaire. (*T*) = *T*-Student. *U* = U–Mann Whitney; (P) = Pearson’s Chi square; (F) = Fisher test. Bold types indicate statistical significance.

### Comparison between groups post-intervention

The ATG proved to be more adherent than the BRG [26]. After the intervention, it showed better results in all variables analyzed, but no significant changes were found between the groups. A large effect size in favor of the ATG in general motivation stands out with a difference of 3.77 points, 95% CI (0.53–7.01), *d* = 0.80. There were also a moderate clinical improvement in favor of the ATG group in extrinsic motivation with a difference of 10.59 points, 95% CI (0.8 to −21.98) (*d* = 0.63); Identified Regulation, 3.89, 95% CI (0.00–7.78), *d* = 0.69; Introjected regulation, 2.03 points, 95% CI (−2.44 to 5.6) (*d* = 0.49), Intrinsic motivation, 17.08 points, 95% CI (−1.27 to 7.75), *d* = 0.64 and higher Amotivation in the BRG with a moderate effect size (*d* = 0.44), a difference of 1.97 points, 95% CI (−5.02 to 1.08) (Table 2).

**Table 2.** Post-intervention ATG vs. BRG.

		**ATG *n* = 17**	**BRG *n* = 18**	**Change (CI 95%)**	**Effect size**	*p* value
Self-efficacy (GSES)	*m* (SD)	42.85 (5.59)	38.06 (9.80)	4.79 (−0.74 to 10.32)	0.60	0.267^U^
Physical activity motivation (BRSQ-36)	*m* (SD)	165.09 (41.61)	162.00 (23.48)	3.09 (4.23 to 26.15)	0.09	0.234^U*^
Extrinsic motivation		65.31 (13.22)	54.72 (19.16)	10.59 (0.8 to −21,98)	0.63	0.073^T*^
Integrated regulation		21.31 (5.59)	16.83 (7.90)	4.48 (−0.25 to 9.21)	0.65	0.127^U*^
Identified regulation		24.00 (3.72)	20.11 (6.99)	3.89 (0.00 to 7.78)	0.69	0.050^T^
Introjected regulation		12.69 (6.67)	10.66 (6.34)	2.03 (−2.44 to 6.5)	0.49	0.474^U^
External regulation		7.18 (4.04)	7.11 (6.07)	0.07 (−3.5 to 3.64)	0.01	0.353^U^
Intrinsic motivation		92.25 (18.37)	75.17 (32.47)	17.08 (−1.27 to 35.37)	0.64	0,180^U^
Knowledge		22.87 (4.81)	17.22 (8.58)	5.65 (−4.12 to 15.42)	0.40	0.093^U^
Stimulation		23.12 (4.59)	18.83 (8.49)	4.29 (−0.49 to 9.07)	0.62	0.335^U^
Accomplish		23.18 (4.53)	19.94 (7.98)	3.24 (−1.27 to 7.75	0.49	0.360^U^
General		22.94 (4.74)	19.17 (4.88)	3.77 (0.53 to 7.01)	**0.80**	0.431^U^
Amotivation		5.31 (2.09)	7.28 (4.13)	−1.97 (−5.02 to 1.08)	−0.44	0.206^U^

BRSQ-36 = Behavioral Regulation in Sport Questionnaire. All analysis were made with *U* Mann–Whitney test was used except those marked with *T* = *T* Student test. (*) = Analysis by comparison of the mean change with the initial measurement was performed as the groups were not similar.

### Comparison between groups at the 3–6 months follow-up

At the 3- and 6-month follow-ups, statistically significant differences in self-efficacy were found in favor of ATG (3-m, *p* = 0.025; 6-m, *p* = 0.007) with a large effect size at 6 months (*d* = 0.79). In terms of physical activity motivation, ATG showed better results in the domain of ‘Extrinsic Motivation’ (3-m, *p* = 0.037, *d* = 0.75; 6-m, *p* = 0.10, *d* = 0.94) and in the domain ‘Identified Regulation’ (3-m, *p* < 0.001, *d* = 1.09; 6-m, *p* = 0.005, *d* = 0.49) after the 3- and 6-month follow-ups. In addition, a large clinical change in external regulation in favor of ATG was found at the 3-month follow-up *d* = 0.84 (Table 3).

**Table 3.** Follow-up 3- and 6- month. ATG vs. BRG.

		**ATG *n* = *17***	**BRG *n* = *18***	**Effect size**	***p* value 3-m**	**ATG *n* = *17***	**BRG *n* = *18***	**Effect size**	***p* value 6-m**
*Self-efficacy (GSES)*	*m* (SD)	44.4 (7.44)	37.8 (9.71)	0.76	0.025	46.17 (5.44)	40.61 (8.30)	0.79	**0.007**
*Physical activity motivation (BRSQ-36)*	*m* (SD)	166.27 (37.94)	138.40 (47.07)	0.05	0.010*	153.82 (39.79)	133,10 (41.44)	0.51	0.006*
Extrinsic motivation		65.93 (10.37)	55.44 (16.64)	0.75	0.037^T*^	64.65 (13.49)	52.01 (13.28)	**0.94**	**0.010^T^**
Integrated regulation		21.90 (7.38)	17.90 (8.41)	0.50	0.149*	20.91 (7.37)	16.20 (7.44)	0.64	0.078
Identified regulation		24.70 (2.59)	19.66 (5.93)	**1.09**	**<0.001**	22.73 (4.69)	20.30 (5.27)	0.49	**0.005**
Introjected regulation		13.81 (6.19)	11 (7.16)	0.42	0.446	13.18 (8.71)	8.9 (6.50)	0.60	0.252
External regulation		8.36 (5.87)	4.80 (1.62)	**0.84**	0.513	7.45 (6.00)	4.5 (1.27)	0.69	0.124
Intrinsic motivation		92.94 (14.08)	77.89 (27.06)	0.69	0.065	109.50 (19.13)	94.17 (29.24)	0.62	0.145
Knowledge		21.45 (7.49)	17.80 (9.21)	0.22	0.135	21.00 (4.60)	17.5 (9.19)	0.19	0.206
Stimulation		23.55 (7.83)	19.60 (8.26)	0.37	0.061	23.06 (4.00)	19.6 (7.86)	0.37	0.206
Accomplish		23.41 (3.78)	20.60 (7.41)	0.32	0.143	23.12 (3.44)	20.72 (4.78)	0.57	0.157
General		23.52 (4.14)	20.16 (7,76)	0.54	0.317	19.91 (8.31)	20.70 (8.93)	−0,09	0.572
Amotivation		4.81 (1.94)	5.30 (2.21)	−0.24	0.55	4.9 (2.07)	5.3 (2.21)	−0.19	0.786

BRSQ-36 = Behavioral Regulation in Sport Questionnaire; all analysis were made with *U*–Mann Whitney test was used except those marked with *T* = *T* Student test. (*) = Analysis by comparison of the mean change with the initial measurement was performed as the groups were not similar.

### Effectiveness of the multimodal program within-groups

An increase in self-efficacy was observed in the ATG group over time, and significant improvements were found at the 6-month follow-up with a large effect size (*p* = 0.016, *d* = 0.79). The same occurred with the domain of ‘Intrinsic Motivation’, for which a significant difference was observed at 6 months (*p* = 0.007, *d* = 1.31). In contrast, overall motivation progressively decreased at 6 months *d* = 1.15 The BRG showed no change in self-efficacy, but after worsening in this variable at the end and at three months follow-up, there was a large improvement. in the domain of ‘Intrinsic Motivation’ at 6 months follow-up with a very large effect size (*p* = 0.012, *d* = 3.36). (Table 4). This group also worsened clinically in general motivation after the intervention *d* = 2.21, maintaining these values at follow-up. But it showed clinical improvements in motivation with very large effect sizes *d* > 1.

**Table 4.** Comparison within groups: ATG and BRG.

**Comparison within the ATG**												
		** Baseline**	**Post-intervention**	**Follow-up 3 months**	**Follow-up 6 months**	***p* value**	**Effect size**	***p* value** Post*	**Effect size**	***p* value** 3 months*	**Effect size**	***p* value** 6 months*
*Self-efficacy (GSES)*	*m* (SD)	40.06 (9.55)	42.85 (5.59)	44.44 (7.44)	46.17 (5.44)	**0.005**	−0.36	0.345	−0.51	0.079	−0.79	**0.016**
*Physical activity motivation (BRSQ-36)*	*m* (SD)	167.24 (18.79)	165.09 (41.61)	166.27 (37.94)	153.82 (39.79)	0.214	0.07		0.03		0.43	
Extrinsic motivation		62.81 (6.37)	65.31 (13.22)	65.93 (10.37)	64.65 (13.49)	0.271	−0.24		−0.29		−0.08	
Integrated motivation		23.25 (2.79)	21.31 (5.59)	21.90 (7.38)	20.91 (7.37)	0.064	0.44		0.24		0.42	
Identified regulation		24.43 (5.15)	24.00 (3.72)	24.70 (2.59)	22.73 (4.69)	**0.015**	0.10	0.479	−0.03	**0.027**	0.17	0.299
Introjected regulation		9.87 (4.06)	12.69 (6.67)	13.81 (6.19)	13.18 (8.71)	0.191	−0.51		−0.66		−0.56	
External regulation		5.25 (1.80)	7.18 (4.04)	8.36 (5.87)	7.45 (6.00)	0.245	**−0.87**		**−1.39**		−0.62	
Intrinsic motivation		96.44 (11.09)	92.25 (18.37)	92.94 (14.08)	109.50 (19.13)	**<0.001^A^**	0.47	0.118	0.39	0.149	**−1.31**	**0.007**
Knowledge		22.75 (5.04)	22.87 (4.81)	21.45 (7.49)	21.00 (4.60)	**0.005**	−0.02	0.426	0.24	0.776	0.32	**0.043**
Stimulation		24.19 (2.40)	23.12 (4.59)	19.60 (8.26)	23.06 (4.00)	**0.034**	0.11	0.531	0.45	0.736	0.08	0.060
Accomplish		24.68 (2.57)	23.18 (4.53)	20.60 (7.41)	23.12 (3.44)	0.052	0.27		0.73		0.42	
General		24.81 (3.31)	22.94 (4.74)	20.16 (7.76)	19.91 (8.31)	**0.008**	0.30	0.086	0.74	**0.031**	**1.15**	**0.023**
Amotivation		5.50 (1.89)	5.31 (2.09)	5.30 (2.21)	4.9 (2.07)	**0.034**	0.10	0.220	0.04	0.076	0.22	0.083
**Comparison within the BRG**												
		**Baseline**	**Post-intervention**	**Follow-up 3 months**	**Follow-up 6 months**	***p* value**	**Effect size**	***p* value** Post*	**Effect size**	***p* value** 3 months*	**Effect size**	***p* value** 6 months*
*Self-efficacy (GSES)*	*m* (SD)	37.39 (7.35)	38.06 (9.80)	37.80 (9.71)	40.61 (8.30)	0.105	−0.08		−0.05		−0.41	
*Physical activity motivation (BRSQ-36)*	*m* (SD)	141.40 (36.38)	162.00 (23.48)	138.40 (47.07)	133,10 (41.44)	0.081	−0.67		0.07		0.21	
Extrinsic motivation		53.38 (12.74)	54.72 (19.16)	55.44 (16.64)	52.01 (13.28)	0.272	−0.08		−0.14		0.11	
Integrated motivation		17.55 (6.89)	16.83 (7.90)	17.90 (8.41)	16.20 (7.44)	0.295^A^	0.10		−0.05		0.19	
Identified regulation		21.11 (5.14)	20.11 (6.99)	19.66 (5.93)	20.30 (5.27)	0.073	0.16		0.26		0.08	
Introjected regulation		10.05 (5.89)	10.66 (6.34)	11.00 (7.16)	8.90 (6.50)	0.168	−0.10		−0.14		0.17	
External regulation		4.66 (1.32)	7.11 (6.07)	4.80 (1.62)	4.50 (1.27)	**0.023**	−0.56	**0.027**	−0.09	**0.05**	0.04	0.076
Intrinsic motivation		84.89 (27.65)	75.17 (32.47)	77.89 (27.06)	94.17 (29.24)	**<0.001**	**2.24**	0.236	**2.45**	0.133	**3.36**	**0.012**
Knowledge		19.66 (7.97)	17.22 (8.58)	17.80 (9.21)	17.50 (9.19)	0.525	0.10		0.01		0.01	
Stimulation		20.17 (7.50)	18.83 (8.49)	19.60 (8.26)	19.60 (7.86)	0.975	0.03		−0.05		0.00	
Accomplish		22.39 (4.94)	19.94 (7.98)	20.60 (7.41)	20.10 (6.40)	0.200	0.49		0.25		0.23	
General		22.66 (8.57)	19.17 (4.88)	20.80 (9.02)	20.70 (8.93)	0.143	**2.21**		**1.97**		**2.11**	
Amotivation		6.44 (4.59)	7.28 (4.13)	5.30 (2.21)	5.30 (2.21)	0.128	**1.48**		**1.79**		**1.16**	

GSES = General Self-Efficacy Scale; MSPSS = Multidimensional Scale of Perceived Social Support; BRSQ-36 = Behavioral Regulation in Sport Questionnaire; A = ANOVA test was used in this analysis.

Friedman test was used in the rest of the analysis. Significance has been calculated by comparing each value with the baseline.

### Correlation between self-efficacy and the other variables in the entire sample before and after the intervention

**Figure 2.**
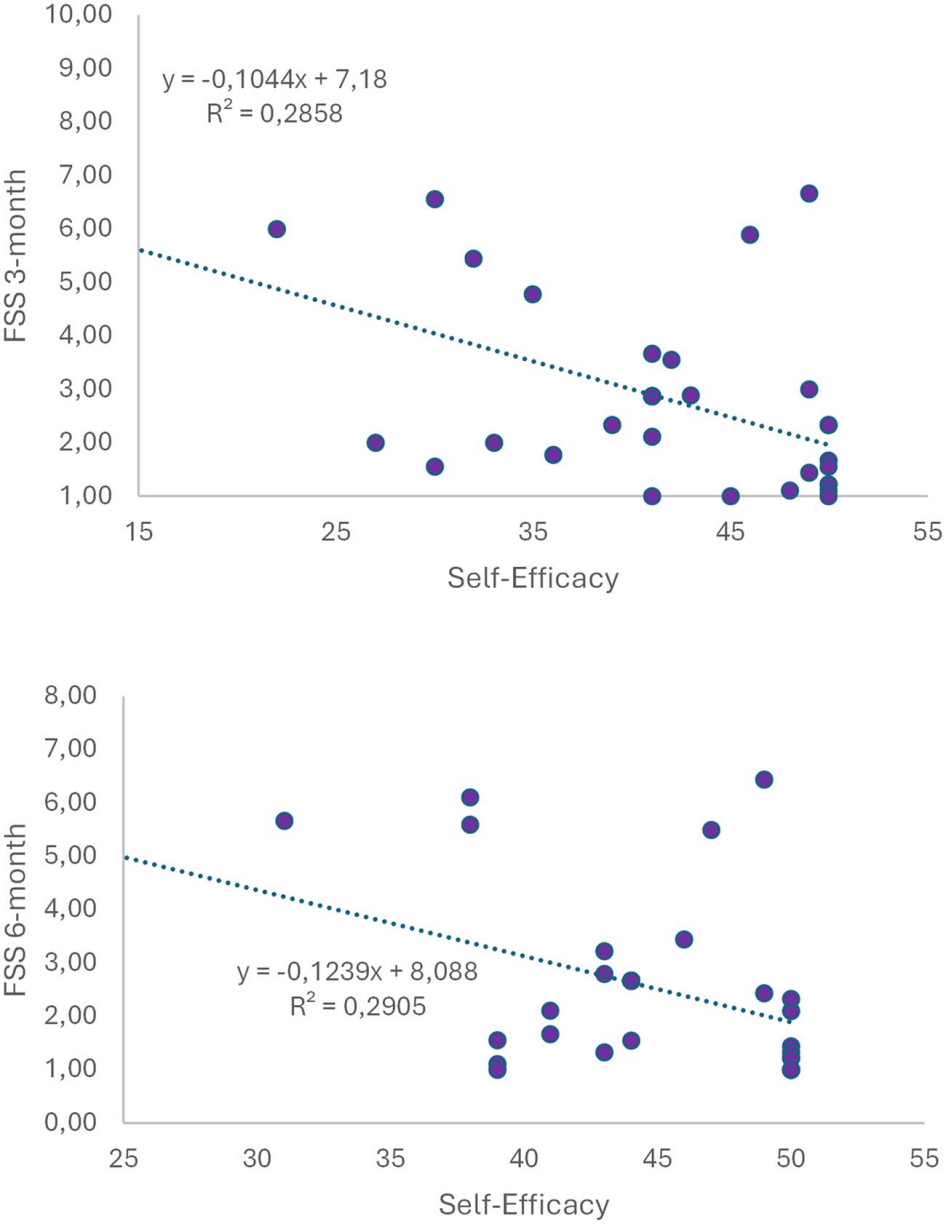
Correlation between self-efficacy and fatigue (FSS) in all patients at 3-month and 6-month follow-up.

There were no significant correlations between self-efficacy and the other variables before the intervention (*p* > 0.05). Although no correlation was found between self-efficacy and the level of fatigue (*p* = 0.510) at the end of treatment, an inverse correlation was found after 3 months with low goodness of fit (*p* < 0.001; *R*^2^ = 0.2858) as well as after 6 months (*p* < 0.001; *R*^2^ = 0.2889), indicating that the higher the level of self-efficacy, the lower the level of fatigue of the patients (Figure 2).

**Figure 3.**
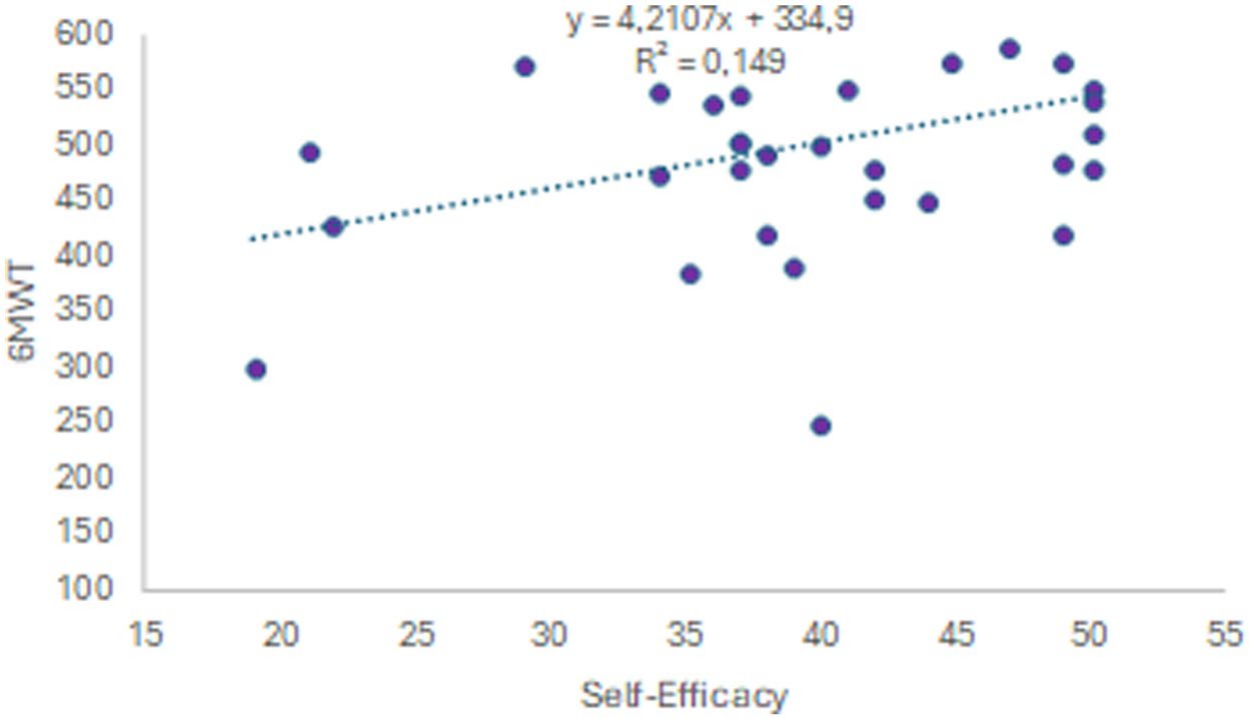
Correlation between self-efficacy and 6-minute walk test (6MWT) in all patients after intervention.

At the end of the intervention, a direct correlation was found between self-efficacy and meters walked in the 6 MWT, although with very low adjustment (*p* = 0.022; *R*^2^ = 0.149) (Figure 3).

A correlation with a low goodness of fit was also found for the 30’ STST and the 30’ ACT (*p* = 0.002, *R*^2^ = 0.261, *p* = 0.017, and *R*^2^ = 0.160, respectively).

No correlation was found between self-efficacy and SF12v2, and a very low correlation was found between self-efficacy and adherence (*p* = 0.029).

## Discussion

This pilot study explores whether a multimodal asynchronous telerehabilitation program can improve motivation and self-efficacy for physical activity in discharged COVID-19 patients, compared to a booklet-based intervention and examines the relationship between self-efficacy and other key factors. Clinical improvements were found in favor of ATG at the end of the intervention in motivation. The ATG group showed at the 3- and 6-month follow-ups improvements in both self-efficacy and extrinsic motivation. BRG only had improvements at 3- and 6 months in some motivation components. A positive correlation was observed after the intervention between self-efficacy and some of the studied variables, including adherence.

The global COVID-19 pandemic requires people to remain indoors, which causes difficulties for patients to follow their prescribed treatments. This led to a significant increase in the development of telecare and telerehabilitation programs. Numerous studies have been conducted to evaluate the effectiveness of these programs. Several studies, including those by Li et al. [3], Pehlivan et al. [4] and Calvo-Paniagua et al. [42] specifically focused on telerehabilitation for COVID-19 survivors, incorporating educational interventions and home-based physical activity, similar to our study. However, none of these studies included measures of perceived self-efficacy or motivation for physical activity, limiting the comparability of our results.

Regarding self-efficacy, no differences were found between groups after treatment. However, changes in favor of ATG were observed at 3 and 6 month-follow-up. These findings align with those of Hawley-Hague et al. [43], who reviewed telerehabilitation self-efficacy in physical therapy and found that patients who received remote physical therapy had higher self-efficacy and motivation [4]. This increased self-confidence contributed to better self-management of their condition. Similarly Cai et al. [44], compared mobile app-based remote home rehabilitation with traditional face-to-face rehabilitation in cardiac ablation patients and found a significant increase in self-efficacy in the experimental group. The patients in our study differed mainly because their hospital situation was severe and in a context of uncertainty, which had an impact on their quality and emotional outcomes [43^,^^45^]. This likely led to a delayed perception of improvement, as recovery can be a more gradual and prolonged process, particularly in patients who have undergone severe illness. The study by [46] showed how patients on COVID-19 mechanical ventilation also showed improvements in self-efficacy after 3 months and progressed over time. These results suggest that telerehabilitation programs may have a more substantial impact on perceived self-efficacy than traditional in-person programs, especially when applied in the medium-term.

Significant differences between the groups were observed in the extrinsic motivation domain in favor of ATG at both the 3- and 6-month follow-ups. In addition to the differences in overall extrinsic motivation scores, significant differences were obtained in the ‘identified regulation’ component, which remained stable over time. Identified regulation refers to a type of motivation in which individuals internalize the importance of physical activity, aligning it with their personal goals and values, which motivates them to engage in the activity even if it is not inherently enjoyable [47]. By identifying with an activity, individuals consciously integrate it into their values, thereby enhancing their perceived autonomy [48,49]. Regarding intrinsic motivation, no significant differences were found between groups. However, a trend of better results can be observed in favor of ATG for all components of intrinsic motivation. These differences could be clinically relevant, so further studies analyzing the effects of rehabilitation programs on intrinsic motivation would be necessary to confirm this finding. This type of motivation is driven by the pleasure that individuals experience when learning, with enjoyment serving as the main motivator for their actions [50].

After the intervention, a positive correlation was found between self-efficacy and various variables studied, including fatigue level, physical condition, and adherence. However, no significant correlation was found between self-efficacy and QoL in this study. Self-efficacy is closely associated with overcoming barriers to exercise and is a key predictor of physical activity [51–53]. Our findings are consistent with this, showing that as self-efficacy increases, barriers to exercise decrease and better adherence is promoted, achieving the observed correlations and improvements in physical condition. Regarding self-efficacy and its impact on fatigue, most studies have focused on cancer patients. For example, Poort et al. [54], found that higher self-efficacy significantly reduced fatigue severity in patients with advanced cancer who experienced fatigue. Similarly Akin & Kas Guner reported that higher self-efficacy positively influences both fatigue and quality of life. Although our study did not replicate this effect on quality of life, a similar trend in self-efficacy was observed at the 3- and 6-month follow-up. It is possible that the positive impact of self-efficacy on fatigue was mediated by improved adherence to treatment. Although a very low correlation between self-efficacy and adherence was found in our study, preliminary published results indicated that adherence was higher in the ATG group than in the BGR group. This suggests that better adherence to telerehabilitation programs may reduce fatigue and improve overall fitness. Therefore, our results are consistent with the literature and confirm the importance of the perception of self-efficacy for adherence to the treatments prescribed by health professionals, and thus, for the achievement of the resulting benefits. Rehabilitation programs should consider this variable in their planning and implementation.

The present study emphasizes the importance of assessing self-efficacy and motivation in clinical practice, as these variables significantly influence treatment adherence []. These variables are not only interrelated but also mutually reinforcing; improving them can lead to better outcomes for patients, including greater treatment engagement and lower likelihood of non-adherence.

In healthcare settings, validated scales to measure self-efficacy and motivation can be integrated into the initial assessment to identify areas in need of improvement and develop tailored interventions. The study by Ng et al. [55] suggests that self-efficacy and motivation are improved by role modeling, promoting autonomy, building confidence, providing information, setting goals, providing feedback, and consistent support.

The implementation of these interventions faces challenges such as technical limitations, the digital divide, and data security. Tailored interventions and a hybrid approach (in-person and remote sessions) can address these issues. Adequate training of professionals is crucial to ensure effective and safe use, and to maintain high-quality care.

## Clinical implications

The expansion of telerehabilitation after the COVID-19 pandemic removed many barriers to its widespread adoption, including resistance to change, low technological self-efficacy and limited funding for remote visits[56,57]. Despite the challenges of this transition, telerehabilitation has not only reduced unnecessary in-person sessions, but has also enabled earlier access to specialized care, reduced the burden of patient transport, and increased comfort for both patients and caregivers while minimizing the risk of infectious disease transmission.

However, the ongoing uncertainty about the effectiveness of telerehabilitation could lead physical therapists to discontinue these services, jeopardizing the momentum gained during the pandemic.

Integrating telehealth into the patient care infrastructure today is critical to creating a more resilient and sustainable healthcare system that can withstand future global health crises. Consequently, telerehabilitation should become the standard rather than the exception, and the development and support of innovative technologies should be encouraged. Therefore, this study highlights the crucial role of motivation and self-efficacy in maintaining effective programs. Only by addressing these factors can we develop effective interventions that improve access to care for people with chronic diseases.

### Strengths and limitations

This study is a secondary analysis of a randomized pilot trial, presenting preliminary data that suggests promising outcomes for a multimodal program incorporating therapeutic exercise and health education for post-COVID-19 patients. Notably, this study is among the few to examine psychosocial variables in conjunction with emerging technologies. We also conducted a thorough analysis of self-efficacy, a critical factor that should be considered in the planning and implementation of rehabilitation programs. Our findings contribute to a better understanding of the relationship between self-efficacy and adherence to prescribed exercises, particularly in the context of telerehabilitation.

However, there is a risk of low power inherent to a pilot study. Due to design limitations, and the sample limited to a specific geographical area, these results cannot be generalized; further, replication and larger studies are needed to validate these exploratory findings. Socio-demographic variables such as level of education or technology literacy that could influence the result were also not collected.

## Conclusions

In conclusion, this study provides preliminary evidence supporting the effectiveness of a multimodal telerehabilitation program to enhance self-efficacy and extrinsic motivation for physical activity in post-acute COVID-19 patients, regarding the identified regulation component. These findings highlight the potential of asynchronous telerehabilitation to improve self-efficacy and extrinsic motivation in a short period of time, which could facilitate better adherence to rehabilitation programs and improve physical conditions. However, the results seem to be limited in the long term. Owing to the pilot nature and design limitations, these results should be interpreted with caution. Further research with larger sample sizes is necessary to validate these findings and to explore their generalizability to broader population groups.

## Data Availability

The datasets generated and/or analyzed during the current study are available from the corresponding author upon reasonable request.
